# Psychometric properties of the Curiosity and Exploration Inventory-II among Kenyan adolescents

**DOI:** 10.3389/fsoc.2023.1189915

**Published:** 2023-11-22

**Authors:** Natalie E. Johnson, Daisy Nerima, Ngina Kahura, Tom L. Osborn

**Affiliations:** ^1^Shamiri Institute, Nairobi, Kenya; ^2^Division of Clinical Epidemiology, Department of Clinical Research, University Hospital Basel, Basel, Switzerland; ^3^Department of Clinical Research, University of Basel, Basel, Switzerland

**Keywords:** curiosity, character strengths, mental health, wellness, sub-Saharan Africa, measurement, Kenya

## Abstract

**Introduction:**

Curiosity is a fundamental trait that drives exploration, motivation, learning, and growth. However, research on this character strength in sub-Saharan African populations is very scarce. To address this gap in the literature, we sought to determine the psychometric properties of the Curiosity and Exploration Inventory- II (CEI-II), a measure for trait curiosity, to provide evidence of validity for its use in research among populations in sub-Saharan Africa. We also aimed to assess for demographic and psychosocial correlates of curiosity among Kenyan high school students.

**Methods:**

A sample of 375 participants in Kenya completed the CEI-II, as well as demographic information on sex, age, form in school, psychosocial measures of depression, anxiety, school climate, and social support. Using cross-sectional data, parallel analysis, scree plot, and structural equation modeling were used to determine the factor structure of the CEI-II among the Kenyan adolescent population.

**Results:**

A one-factor solution was found to be the best fitting model, differing from the two-factor structure found in the original development of the measure. Internal consistency, convergent and discriminant validity, and predictors of trait curiosity were also examined. The CEI-II demonstrated good internal consistency and convergent validity with social support from family, friends, significant others, and school climate. Discriminant validity was demonstrated by the non-significant correlation between curiosity and depression. A hierarchical regression model showed that curiosity was significantly predicted by social support from family, significant others, school climate, and anxiety, with males being more curious than females.

**Discussion:**

The CEI-II is a valid measurement tool to capture trait curiosity in Kenyan adolescents, and our findings provide insight into the relationship between curiosity and other psychosocial factors in this population.

## 1 Introduction

Historically, one of the fundamental goals of psychology was enabling individuals to flourish and thrive. However, this took a backseat to a focus on assessing and managing psychological disorders (Joseph and Wood, 2010). The turn of the century brought with it a rekindling of psychology’s need to develop its knowledge and practices on the flourishing of an individual. This led to the rise of the field of positive psychology, which is the study of positive experiences, positive individual traits and the institutions that facilitate their development. Positive psychology seeks to establish a scientific understanding of the psychology of positive human functioning and interventions that encourage individuals and communities to thrive (Seligman and Csikszentmihalyi, 2000; Park et al., 2004; Lee Duckworth et al., 2005; Ruch et al., 2014).

Recent progress in the field of positive psychology has resulted in the emergence of the concept of character strengths, which are typically classified under six universal virtues: wisdom, courage, humanity, justice, temperance, and transcendence (Dahlsgaard et al., 2005). Character strengths are a subset of personality traits that have a moral connotation, are valued across cultures and historical periods, and reflect the six universal virtues. For instance, curiosity is a psychological manifestation of the virtue of wisdom (Peterson and Seligman, 2004; Kaczmarek et al., 2014).

Research suggests that character strengths are associated with positive outcomes in various areas of life, including well-being, life satisfaction, and self-efficacy (Park et al., 2004; Seligman et al., 2005). Among adolescents, character strengths have been found to predict and contribute to well-being, global life satisfaction, and self-efficacy. Furthermore, intellectual strengths, such as curiosity, have been shown to predict higher levels of satisfaction during adolescence. Character strengths have also been found to negatively correlate with behavioral and psychological problems in adolescents (Gillham et al., 2011; Ruch et al., 2014).

The practice of character strength has been shown to be effective to broaden positive affect and reduce negative states while increasing well-being (Sin and Lyubomirsky, 2009). When individuals employ and develop their character strengths in daily activities, it produces an immediate invigoration and in the long term, provides a sense of authenticity that leads to improvements in well-being and reduced depressive symptoms (Kaczmarek et al., 2014). Therefore, character strength interventions can be valuable and impactful tools to improve adolescent well-being and reduce psychopathology (Gillham et al., 2011; Venturo-Conerly et al., 2022).

Recent research from Kenya has shown that character strength interventions can be effective in reducing depressive symptoms for Kenyan adolescents (Osborn et al., 2020a, 2021; Venturo-Conerly et al., 2022). A recent study with Kenyan high school students demonstrated that the effects of a single session of a digital character strengths intervention exceeded those of traditional psychotherapy (Osborn et al., 2020b). It seems, therefore, that positive psychology interventions present an approach to adolescent psychopathology that is particularly beneficial for low-resource settings (e.g., Kenya and sub-Saharan Africa) because they help bypass the contextual mental health stigma and are less resource-intensive than traditional interventions (Osborn et al., 2020a, 2021; Campbell and Osborn, 2021; Venturo-Conerly et al., 2022). More research is needed to expand our understanding of character strengths and their potential benefits for adolescent mental health in low-resource settings.

Curiosity is one of the most common character strengths and is a fundamental concept of psychology. Interest in it as a psychological construct began in the mid-twentieth century and it has since evolved in definition, conceptualization, and assessment (Berlyne, 1954; Reio et al., 2006). Currently, curiosity is viewed as a positive emotional-motivational system that drives individuals to seek out and embrace new knowledge and experiences (Kashdan et al., 2004, 2009). It is an essential intellectual strength that drives learning and exploration of novel and challenging ideas to build knowledge and competence (Kashdan and Silvia, 2009; Kaczmarek et al., 2014; Kidd and Hayden, 2015).

Curiosity is regarded as a positive emotional vital sign of an individual’s well-being (Spielberger, 2006; Spielberger and Reheiser, 2009). It is also strongly associated with intrinsic motivation, interest and flow, which form the optimal states of psychological functioning. Individuals with higher levels of curiosity initiate behaviors that enhance their personal growth, and increase their tolerance of uncertainty and challenging situations (Kashdan et al., 2004; Gallagher and Lopez, 2007). Further, seeking novelty and challenge (driven by curiosity) leads to more sustainable increases in well-being than pleasure-focused approaches and is associated with greater meaning in life (Kashdan and Steger, 2007).

Curiosity may function as a protective buffer against poor psychological outcomes because of its capacity to increase well-being. Higher levels of curiosity are associated with lower levels of depression, and lower levels of curiosity can result in poor tolerance of uncertainty which predisposes an individual to anxiety disorders (Spielberger, 2006; Kashdan et al., 2009; Kaczmarek et al., 2014). Academically, curiosity is also important because students with greater curiosity have been shown to have greater success than their less curious peers (Kashdan and Silvia, 2009). Curiosity is a concept that holds a lot of promise to enable individuals and communities to flourish, necessitating an increase in knowledge and research on this core character strength.

To advance the understanding of curiosity and its clinical uses, valid and reliable measures of curiosity are essential. Multiple measures have been developed to gain insight into the structure and components of the construct. However, challenges exist that limit our ability to adequately assess for curiosity. First, some measures are limited by focusing on the objects of curiosity rather than its intrinsic qualities, thus failing to capture the full breadth of the construct. Second, domain-specific measures may not adequately capture the heterogeneity of curiosity that is impacted by varying interests. Additionally, activity-based scales are prone to high item-specific error (Kashdan et al., 2009). Lastly, some measures include items that assess positive affect, which is not a fundamental part of the construct of curiosity (Kashdan et al., 2004; Wagstaff et al., 2021).

The Curiosity and Exploration Inventory (CEI) was designed to accurately capture the defining features of curiosity. It is a general-use scale that is not specific to any particular activity or topic, which ensures that the construct of curiosity is adequately evaluated (Kashdan et al., 2004). The scale was subsequently refined to create the Curiosity and Exploration Inventory-II (CEI-II), which is a brief, reliable and valid measure of curiosity that expands the breadth of the construct (Kashdan et al., 2009). This was achieved by including items that assess the willingness to manage the tension that arises from encountering novelty and/or uncertainty, which is an important facet of curiosity that requires assessment. Additionally, the original subscales of the CEI (exploration and absorption) were modified, with the absorption subscale being dropped due to poor psychometric properties, and the subscales being adapted to stretching and embracing.

The CEI-II has demonstrated adequate psychometric properties among college students in North America, Europe, and Asia (Kashdan et al., 2009; Ye et al., 2015; Balgiu, 2018; Setyowati et al., 2020), as well as secondary school students in Europe (Jovanovic and Brdaric, 2012; Jovanović and Gavrilov-Jerković, 2014). However, the CEI-II has not yet been validated for use with sub-Saharan African populations. Additionally, the demographic profile of trait curiosity and its relationship with other mental health constructs has yet to be explored among this population. Thus, the current study aimed to evaluate the psychometric properties of the CEI-II among Kenyan adolescents and explore the correlates of trait curiosity in this population. This study is the first of its kind to investigate curiosity among sub-Saharan Africans.

## 2 Methods

### 2.1 Participants

A sample of 375 Kenyan secondary school students was recruited from a school in Kibera, a densely populated urban informal settlement in Nairobi. Their ages ranged from 13 to 20 (*M* = 17.08, SD = 1.28). The students were enrolled in a classroom-based arts and literacy program called Pre-texts. This cross-sectional study was conducted using the baseline data point from the larger Pre-texts study.

### 2.2 Procedure

Ethical approval for this study was obtained from the Kenyatta University Ethical Review Committee (Protocol number PKU/2561/E1687). All students in the school were invited to voluntarily participate in the study via the school administration, and parental consent was sought via a letter explaining the study. Informed consent or assent was then sought directly from participants prior to enrollment in the study. The participants completed a paper-based survey which included demographics of sex, age, and form, as well as measures of curiosity, wellbeing, mental health, social support, and school climate.

### 2.3 Measures

#### 2.3.1 Curiosity

The CEI-II is designed to measure individual differences in broad dimensions of curiosity (Kashdan et al., 2009). The measure comprises two sub-dimensions, exploration, or stretching, and embracing. It is a 10-item measure that uses a 5-point Likert scale (1 = very slightly or not at all; 2 = a little; 3 = moderately; 4 = quite a bit; 5 = extremely). Because our subjects were adolescents, we were advised by local experts to exclude item 8 of the CEI-II, “I prefer jobs that are excitingly unpredictable.” This measure has shown adequate internal consistency when used with youth and adolescents, with a Cronbach’s α ranging between 0.75 and 0.86 (Kashdan et al., 2009). This study is to the best of our knowledge the first to use this measure among a sub-Saharan African population.

The CEI-II scale used in this study has 9 items. CEI-II_1 measures seeking information, CEI-II_2 measures enjoying uncertainty, CEI-II_3 measures embracing challenges, CEI-II_4 measures looking for new things, CEI-II_5 measures learning from challenges the item CEI-II_6 measures liking frightening things, CEI-II_7 measures looking for challenges, CEI-II_8 measures seeking opportunities, and CEI-II_9 measures embracing new things.

#### 2.3.2 Wellbeing

The Short Warwick-Edinburgh Wellbeing Scale (SWEMWBS) was used to measure wellbeing (Ng Fat et al., 2017). The SWEMWBS is a 7-item shortened version of the Warwick-Edinburgh Mental Wellbeing Scale, a 14-item tool developed for public mental health monitoring and assessment (Tennant et al., 2007). The SWEMWBS is often used in psychological surveys and interventions due to its lower participant burden (Tennant et al., 2007; Ng Fat et al., 2017; Shah et al., 2018; Koushede et al., 2019). This measure has been used with Kenyan adolescents and has shown adequate internal consistency (Cronbach’s *α* = 0.78) (Osborn et al., 2020b).

#### 2.3.3 Anxiety

The Generalized Anxiety Disorder Screener-7 item (GAD-7) is a measure used globally to screen for generalized anxiety disorder in adolescents and adults (Spitzer et al., 2006). The GAD-7 has shown good internal consistency when used with Kenyan adolescents (Cronbach’s *α* = 0.78) (Osborn et al., 2020a).

#### 2.3.4 Depression

We used the Patient Health Questionnaire-8 (PHQ-8) to measure symptoms of depression. The PHQ-8 is the 8-item version of the PHQ-9 and omits the question which assesses for suicidality (Kroenke et al., 2001; Kroenke and Spitzer, 2002). PHQ-8 scores are highly correlated with PHQ-9 scores, and the same cutoffs can be used to assess depression severity (Kroenke et al., 2001, 2009). The PHQ-8 has been validated for use in Kenyan adolescents (Cronbach’s *α* = 0.73) (Osborn et al., 2020a). The ninth item was omitted due to expressed discomfort on behalf of school affiliates and local experts.

#### 2.3.5 Social support

The Multidimensional Scale of Perceived Social Support (MSPSS) is designed to measure satisfaction with social support (Zimet et al., 1988). It consists of three subscales: the “friends” subscale, which measures support from friends, the “family” subscale which measures support from family, and the significant others subscale, which measures support from significant others (Osborn et al., 2020c, 2022). The MSPSS has demonstrated adequate internal consistency when used with Kenyan adolescents (Cronbach’s *α* = 0.86–0.88; Osborn et al., 2020a, 2022).

#### 2.3.6 School climate

The School Climate Measure (SCM) is a comprehensive measure of school climate (Zullig et al., 2021). This questionnaire consists of 10 domains, including positive student-teacher relationships, order and discipline, opportunities for student engagement, school physical environment, academic support, parental involvement, school connectedness, perceived exclusion/privilege, school social environment, and academic satisfaction. The SCM has been shown to correlate with school satisfaction, life satisfaction, and quality of life (Zullig et al., 2018).

The SCM has demonstrated convergent and discriminant validity when tested with adolescents in North America (Zullig et al., 2018). Local experts and school affiliates selected 13 items from the larger SCM which they deemed particularly impacted the school climate of Kenyan secondary schools. The version of the SCM used in this study is called the School Climate Measure for Kenyan Adolescents (SCM-KA). The SCM-KA demonstrated good internal consistency (Cronbach’s *α* = 0.85).

#### 2.3.7 Demographic characteristics

Participants also self-reported their demographic information. Participants provided their age, gender, and form (year in school). This was done to assess the demographic profile of trait curiosity.

### 2.4 Statistical analysis

Internal consistency of all measures was assessed using Cronbach’s α and Greatest Lower Bound α (GLB α). Measures with Cronbach’s α and GLB α scores of 0.70 or higher were used in subsequent analysis, while those with scores less than 0.70 were not considered.

Demographic characteristics were summarized using frequencies and percentages. Means and standard deviations were computed for CEI-II scores per demographic characteristic, and *t*-tests were used for two-group comparisons, while ANOVA was used for comparisons across more than two groups.

Before conducting validity tests, data requirements for factor analysis were checked using the Kaiser-Meyer-Olkin (KMO) and Bartlett’s Sphericity tests (Li et al., 2020). The data met the requirement for factor analysis, as evidenced by a KMO value of 0.84 and a Bartlett’s significance value less than 0.05.

Parallel analysis was performed to determine the possible number of factors in the data (Li et al., 2020), accompanied by a scree plot of eigenvalues (Willmer et al., 2019). The number of factors in the data was equal to the number of factors with eigenvalues greater than one (Golub and van der Vorst, 2000). Standardized factor loadings were assessed across the top possible factors.

Three models were identified based on their well-established validity and applicability to our research problem (Kaczmarek et al., 2014; Ye et al., 2015; Balgiu, 2018). A one-factor solution groups all the items in the CEI-II measure as one. In the case of two factors, the items are grouped into two components: embracing or exploration. A three-factor solution dichotomizes the embracing component and thus results in three components.

Structural Equation Modeling (SEM) was used to identify the best fitting model. Three models were fitted using maximum likelihood estimation (MLR) and assessed using the Tucker-Lewis Index (TLI), Comparative Fit Index (CFI), Root Mean Square Error of Approximation (RMSEA), and Bayesian Information Criterion (BIC) (Rosseel, 2012). These models were the single-factor, two-factor, and three-factor models, all of which have been used in previous studies of the CEI-II in other contexts.

The estimator for all models was specified as the MLR and the missing value method was specified to full information maximum likelihood (FIML). FIML is a technique that estimates parameters while considering missing data in a way that maximizes the use of available information. Lastly, to prevent overfitting, the ridge parameter within the SEM function was used (Jacobucci et al., 2019).

TLI and CFI scores of ≥0.9 indicate an acceptable fit, scores of ≥0.95 indicate a very good fit; RMSEA values of no greater than 0.05 indicate a good fit, values between 0.05 and 0.08 indicate moderate fit, values of greater than 0.08 indicate a poor fit (Shi et al., 2019). Models with lower BIC are usually preferred (Penny et al., 2007).

We also investigated the discriminant validity of the CEI-II score (Hubley, 2014). To assess this, we tested whether other variables were negatively or positively correlated with the CEI-II score. Pearson’s correlation was used to investigate the correlation between study variables (Schober et al., 2018).

Lastly, we assessed the association between the curiosity measure and other psychosocial and demographic variables with hierarchical multiple regression analysis (Petrocelli, 2003). This involves adding the predictors (age, gender, social support from family, friends and significant other, school climate, depression, and anxiety) stepwise into the model. Estimated beta values and 95% confidence intervals were reported for the predictors in the models. A *p*-value of less than 0.05 was considered statistically significant. All analyses were conducted using *R* version 4.2.2 (R Core Team, 2021). The “lavaan,” “semPlot,” and “semTools” packages were used (Rosseel, 2012; Epskamp, 2015; Jorgensen et al., 2016).

## 3 Results

### 3.1 Internal consistency

Measures for curiosity (Cronbach’s *α* = 0.74, GLB *α* = 0.79), depression (Cronbach’s *α* = 0.67, GLB *α* = 0.74), anxiety (Cronbach’s *α* = 0.77, GLB *α* = 0.81), social support (Cronbach’s *α* = 0.88, GLB *α* = 0.93), and school climate (Cronbach’s *α* = 0.85, GLB *α* = 0.90) showed good internal consistency and were therefore used in this study. The well-being measure was excluded from further analysis (Cronbach’s *α* = 0.50, GLB *α* = 0.59).

### 3.2 Demographic characteristics

Participants in this study were high school students from Kenya (*n* = 375) aged 13 to 20 years. Table 1 summarizes the demographic characteristics of the participants according to their CEI-II scores. Most participants (32.09%) were in Form 3, with 28.88, 25.94, and 13.10% from Form 2, 4, and 1, respectively. Students in Form 2 had a slightly higher CEI-II mean score (30.99 ± 6.05); however, the differences in means were not significant (*f* = 0.63, *p* = 0.22). Older adolescents aged 17–20 years had a relatively higher CEI-II mean score (30.29 ± 7.38) compared to younger adolescents (29.59 ± 6.58) aged 13–16 years although the differences in the means were not statistically significant (*t* = −0.84, *p* = 0.40). Males had a slightly higher CEI-II mean score (30.69 ± 6.94) compared to females (29.49 ± 7.31); even so, the means were not statistically different (*t* = −1.49, *p*-value = 0.14).

**Table 1:** Participant characteristics and demographic distribution of CEI-II scores.

Characteristic	*n* (%)	Mean CEI-II score ± SD
Gender		
Male	189 (50.67)	30.69 ± 6.94
Female	184 (49.33)	29.49 ± 7.31
Form		
1	49 (13.10)	28.46 ± 7.79
2	108 (28.88)	30.99 ± 6.05
3	120 (32.09)	29.64 ± 6.95
4	97 (25.94)	30.60 ± 7.89
Age category		
13–16 years	110 (29.49)	29.59 ± 6.58
17–20 years	263 (70.51)	30.29 ± 7.38

### 3.3 Factor structure evaluation

#### 3.3.1 Factorability of the data

CEI-II had KMO of 0.84, which is above the 0.5 cutoff. The Bartlett’s Sphericity test result was also statistically significant [*χ*^2^ = 480.03, *p* < 0.01, degrees of freedom (df) = 36]. Thus, the CEI-II data met the requirements for factor analysis.

#### 3.3.2 Parallel analysis, scree plot, and factor loadings

**Figure 1:**
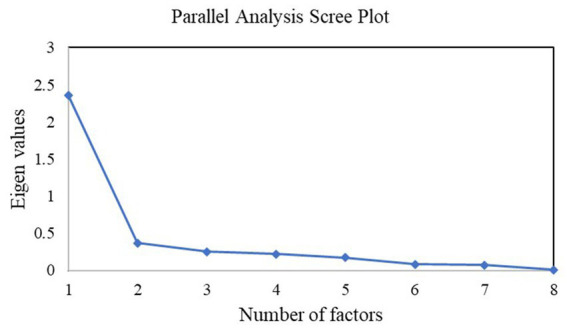
Scree plot; a drop below one in the eigenvalue beyond a single factor suggests a one-factor solution.

Parallel analysis suggested the number of factors and components in the CEI-II measure to be one. Additionally, the scree plot showed that only one factor had an eigenvalue greater than one, as shown in Figure 1. Thus, scree plot and parallel analysis supported a one-factor solution.

We also assessed the factor loadings across all items in the CEI-II. The results revealed that most items were loaded heavily in the first factor compared to the other factors. Particularly, the standardized loading for a one-factor case was 2.36, two-factor was 0.37, and for a three-factor was 0.25. A single-factor solution explained the highest variation of 0.67 in the loadings compared to 0.1 and 0.07 of a two-factor and three-factor solution, respectively. The results suggested that a one-factor solution best explained the curiosity measure as shown in Table 2.

**Table 2:** Results of factor analysis.

	Standardized loading per factor				
CEI-II items	1	2	3	4	5
Seeks information	0.59	0.26	−0.27	−0.12	0.02
Enjoys uncertainty	0.39	0.43	0.16	0.06	−0.13
Embraces challenges	0.52	−0.03	0.09	0.16	−0.02
Looks for new things	0.59	−0.16	−0.15	0.17	0.10
Learns from challenges	0.59	−0.18	−0.08	−0.01	−0.20
Likes frightening things	0.34	0.05	0.26	−0.17	0.19
Looks for challenges	0.57	−0.17	0.21	−0.08	−0.13
Seeks opportunities	0.58	−0.10	0.02	−0.17	0.16
Embraces new things	0.36	0.11	0.10	0.28	0.15
SS loadings	2.36	0.37	0.25	0.22	0.17
Proportion explained	0.67	0.10	0.07	0.06	0.05
Correlation with factors	0.90	0.63	0.55	0.50	0.46
Multiple R square	0.8	0.39	0.3	0.25	0.22

#### 3.3.3 Model comparison

**Figure 2:**
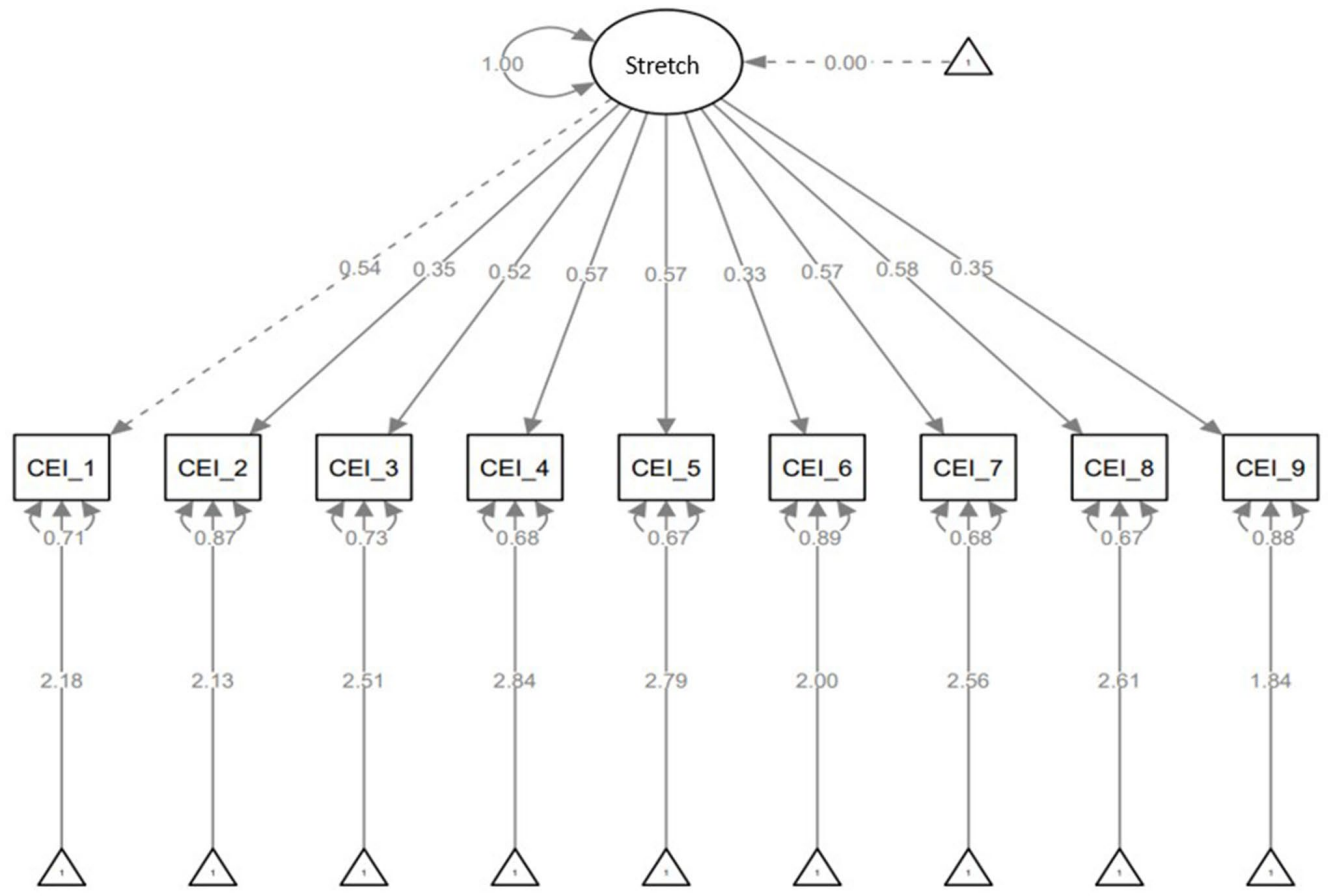
Single-factor model with latent variable of stretching, standardized estimates, residuals, and variances.

The latent variables vary as per the models with single, two, and three-factor models having one, two, and three latent variables, respectively. In the single-factor model, the latent variable is stretch, and the observed variables are the nine items from the CEI-II scale. Figure 2 shows the relationship between the latent and the observed variables. All the CEI-II items are positively related to stretching. Stronger relationships between stretch and CEI-II_1,3,4,5,7, and 8 (strength of relationships: CEI-II_1 = 0.54, CEI-II_3 = 0.52, CEI-II_4 = 0.57, CEI-II_5 = 0.57, CEI-II_7 = 0.57, CEI-II_8 = 0.58, and CEI-II_9 = 0.35) and less strong relationships between stretch and CEI-II_2,6, and 9 (strength of relationships: CEI-II_2 = 0.35, CEI-II_6 = 0.33, and CEI-II_9 = 0.35).

**Figure 3:**
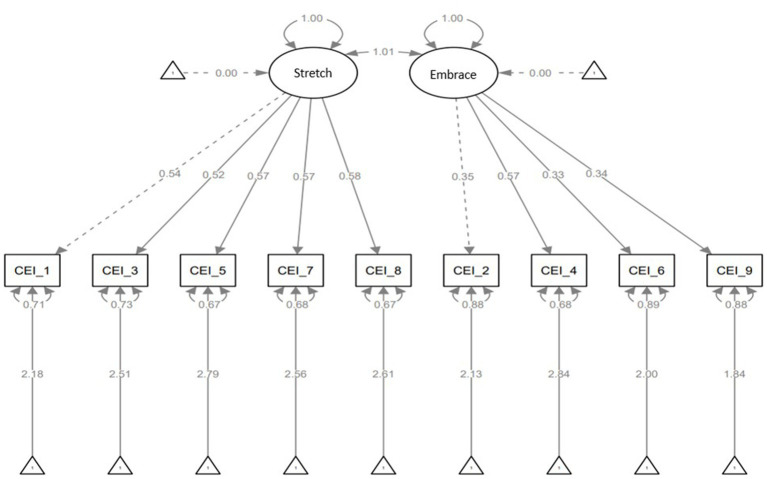
Two-factor model with latent variables of stretching and embracing, and standardized estimates, residuals, and variances.

In the two-factor model, the CEI-II scale has two latent variables; stretch and embrace. Stretch has five observed variables (CEI-II_1,3,5,7 and 8) and embrace has four observed variables (CEI-II_2,4,6, and 9). Figure 3 shows the relationship between the latent and the observed variables. There is a strong relationship between stretch and CEI-II_1,3,5,7, and 8 (strength of relationships: CEI-II_1 = 0.54, CEI-II_3 = 0.52, CEI-II_5 = 0.57, CEI-II_7 = 0.57, and CEI-II_8 = 0.58). There is also a strong relationship between embrace and CEI-II_4 (strength of relationship: CEI-II_4 = 0.57) and less strong relationships between embrace and CEI-II_2,6, and 9 (strength of relationships: CEI-II_2 = 0.35, CEI-II_6 = 0.33, and CEI-II_9 = 0.34).

**Figure 4:**
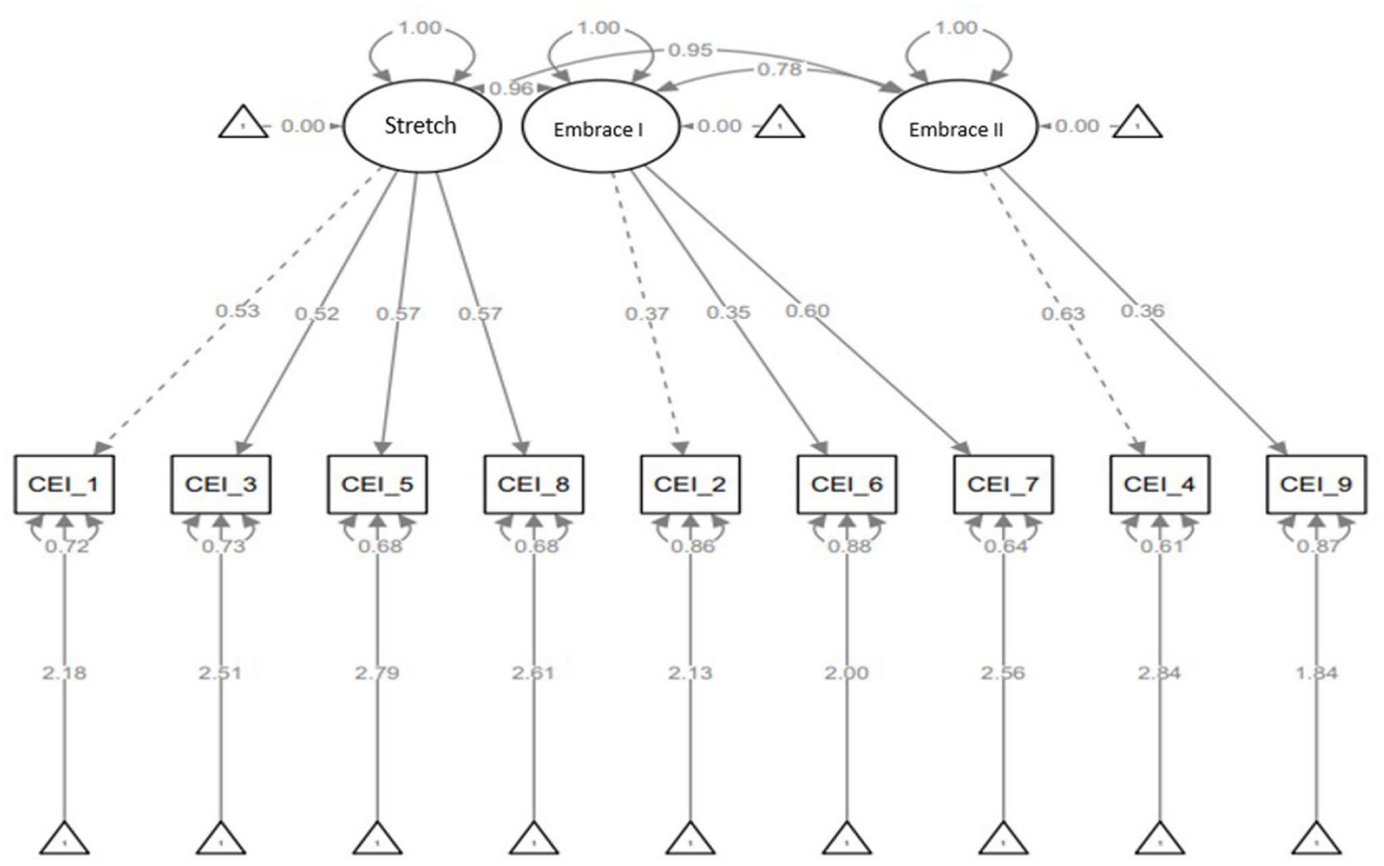
Three-factor model with latent variables of stretching, embracing I and II, and standardized estimates, residuals, and variances.

In the three-factor model, the CEI-II scale has three latent variables; stretch, embrace I and and II. Stretch has four observed variables (CEI-II_1,3,5, and 8), embrace I has three observed variables (CEI-II_2,6, and 7), and embrace II has two observed variables (CEI-II_4 and 9). Figure 4 shows the relationship between the latent and the observed variables. There is a strong relationship between stretch and CEI-II_1,3,5, and 8 (strength of relationships: CEI-II_1 = 0.53, CEI-II_3 = 0.52, CEI-II_5 = 0.57, and CEI-II_8 = 0.57). There is also a strong relationship between embrace I and CEI-II_7 (strength of relationship: CEI-II_7 = 0.60) and less strong relationships between embrace I and CEI-II_2 and 6 (strength of relationships: CEI-II_2 = 0.37 and CEI-II_6 = 0.35). Furthermore, there is a strong relationship between embrace II and CEI-II_4 (strength of relationship: CEI-II_4 = 0.63) and a less strong relationship between embrace II and CEI-II_9 (strength of relationship: CEI-II_9 = 0.36).

The results for the TLI, CFI, RMSEA, and BIC for the models are shown in Table 3. Although all models performed well, a single-factor solution was the best-fitting model, as it had the lowest BIC (*χ*^2^ = 29.38, TLI = 0.99, CFI = 0.99, RMSEA = 0.01, BIC = 9852.41). The second-best fitting model was the two-factor solution (*χ*^2^ = 29.33, TLI = 0.99, CFI = 0.99, RMSEA = 0.02, BIC = 9858.13), followed by a three-factor solution (*χ*^2^ = 26.28, TLI = 0.99, CFI = 0.99, RMSEA = 0.02, BIC = 9866.62).

**Table 3:** Structural equation modeling for model comparison.

Model	*χ* ^2^	Df	*p*-value	TLI	CFI	RMSEA [95%]	BIC
Single factor	29.38	27	0.34	0.99	0.99	0.01 [0.00, 0.04]	9852.41
Two factors	29.33	26	0.30	0.99	0.99	0.02 [0.00, 0.05]	9858.13
Three factors	26.28	24	0.34	0.99	0.99	0.02 [0.00, 0.05]	9866.62

### 3.4 Correlations between the study variables

Curiosity was found to be significantly and positively correlated with support from family (*r* = 0.31, CI = [0.20, 0.41], *p* < 0.01), support from friends (*r* = 0.25, CI = [0.15, 0.35], *p* < 0.01), support from significant others (*r* = 0.31, CI = [0.20, 0.41], *p* < 0.01) and school climate (*r* = 0.41, CI = [0.31, 0.50], *p* < 0.01). There was very slight and non-significant correlation between depression and curiosity (*r* = 0.05, CI [−0.07, 0.17], *p* = 0.99). Also, the curiosity measure was very slightly correlated with anxiety (*r* = 0.03, CI [−0.09, 0.15], *p* = 0.99), though this relationship was not statistically significant.

Depression positively correlated with anxiety (*r* = 0.69, CI = [0.62, 0.75], *p* < 0.01) and negatively correlated with social support from family (*r* = −0.23, CI = [−0.34, −0.12], *p* < 0.01), friends (*r* = −0.21, CI = [−0.32, −0.10], *p* < 0.01), significant others (*r* = −0.21, CI = [−0.32, −0.10], *p* < 0.01) and school climate (*r* = −0.31, CI = [−0.42, −0.22], *p* < 0.01). Also, anxiety was negatively correlated with social support from family (*r* = −0.29, CI = [−0.39, −0.18], *p* < 0.01), friends (*r* = −0.21, CI = [−0.32, −0.10], *p* < 0.01), significant others (*r* = −0.24, CI = [−0.35, −0.13], *p* < 0.01) and school climate (*r* = −0.31, CI = [−0.42, −0.20], *p* < 0.01). There was a positive correlation between school climate and social support from family (*r* = 0.50, CI = [0.41, 0.58], *p* < 0.01), friends (*r* = 0.43, CI = [0.33, 0.52], *p* < 0.01) and significant others (*r* = 0.30, CI = [0.19, 0.40], *p* < 0.01). Table 4 shows the summary of the correlations between the study variables.

**Table 4:** Correlations between the study variables.

Variable	1	2	3	4	5	6	7
1. Age							
2. Curiosity	0.05 [−0.06, 0.16]						
3. Depression	0.07 [−0.05, 0.18]	0.05 [−0.07, 0.17]					
4. Anxiety	0.06 [−0.05, 0.18]	0.03 [−0.09, 0.15]	0.69** [0.62, 0.75]				
5. Family support	−0.02 [−0.13, 0.09]	0.31** [0.20, 0.41]	−0.23** [−0.34, −0.12]	−0.29** [−0.39, −0.18]			
6. Friends support	−0.01 [−0.12, 0.09]	0.25** [0.15, 0.35]	−0.21** [−0.32, −0.10]	−0.21** [−0.31, −0.10]	0.42** [0.32, 0.50]		
7. Significant other support	0.06 [−0.05, 0.17]	0.31** [0.20, 0.41]	0.21** [−0.32, −0.10]	−0.24** [−0.35, −0.13]	0.57** [0.49, 0.64]	0.56** [0.48, 0.63]	
8. School climate	0.03 [−0.08, 0.14]	0.41** [0.31, 0.50]	−0.31** [−0.42, −0.20]	−0.31** [−0.42, −0.20]	0.50** [0.41, 0.58]	0.43** [0.33, 52]	0.30** [0.19, 0.40]

Values in square brackets indicate the 95% confidence interval for each correlation. The confidence interval is a plausible range of population correlations that could have caused the sample correlation (Cumming, 2014). * Indicates *p* < 0.05. ** indicates *p* < 0.01. Bolded values indicate a statistically significant *p*-value.

### 3.5 Hierarchical multiple regression analysis

We conducted hierarchical multiple regression analysis to determine the significant predictors of the CEI-II score. In the first step, we introduced age and gender to the model. In the second step, we added social support from family, friends, and significant others. Thirdly, school climate was added, and fourth anxiety. Lastly, depression was added to the model. The findings are summarized in Table 5.

**Table 5:** Hierarchical multiple regression analysis.

Variable	Estimate [95%CI]	*p*-value	*R* ^2^	Δ*R*^2^	*F*
Step 1			0.04	–	3.53
Gender (Male)	2.74 [0.53, 4.95]	**0.02**			
Age	0.20 [−0.66, 1.06]	0.65			
Step 2			0.20	0.16	8.47
Social support					
Friends	0.17 [−0.64, 0.97]	0.68			
Family	0.80 [0.02, 1.58]	**0.04**			
Significant other	1.27 [0.40, 2.15]	**<0.01**			
					
Step 3			0.24	0.04	8.54
School climate	0.17 [0.04, 0.29]	**0.01**			
Step 4			0.28	0.04	9.35
Anxiety	0.36 [0.15, 0.57]	**<0.01**			
Step 5			0.29	0.01	8.39
Depression	0.16 [−0.10, 0.43]	0.23			

Bolded values indicate statistically significant values at *p* < 0.05. Values in square brackets indicate the 95% confidence interval for each estimate.

In the first step, age (*β* = 0.20, *p*-value = 0.65) was not a significant predictor of the CEI-II score whereas gender was significant (*β* = 2.74, *p*-value = 0.02). This model had a relatively low goodness of fit score (*R*^2^ = 0.04, F-statistic = 3.53). Upon adding social support from family (*β* = 0.80, *p*-value = 0.04), friends (*β* = 0.17, *p*-value = 0.68), and significant others (*β* = 1.27, *p*-value < 0.01), the model’s goodness of fit increased by 0.16 (*R*^2^ = 0.20, F-statistic = 8.47). Adding the school climate measure (*β* = 0.17, *p*-value = 0.01) led to an increase in the model’s goodness fit by 0.04 (*R*^2^ = 0.24, F-statistic = 8.54).

Introducing anxiety (*β* = 0.36, *p*-value < 0.01) to the model led to an increase in the model’s goodness of fit by 0.04 (*R*^2^ = 0.28, F-statistic = 9.35). In the last step, we added the depression score to the model. The model’s *R*^2^ increased by 0.01 (*R*^2^ = 0.29, F-statistic = 8.39) and depression was not a significant predictor of curiosity (*β* = 0.16, *p*-value = 0.23). Age, social support from family, social support from significant other, school climate, and anxiety were significant predictors of the CEI-II score. Also, the log-odds of curiosity was predicted to be 2.74 larger for males than for females.

## 4 Discussion

This study demonstrated that the CEI-II is a reliable and valid measure of curiosity among Kenyan adolescents, despite being originally developed with a Western population of undergraduate students (Kashdan et al., 2009). The results suggest that the CEI-II has adequate internal consistency, meaningful correlations with relevant constructs, and a lack of correlation with divergent constructs. However, the two-factor structure of the CEI-II was not supported (Kashdan et al., 2009), and a single-factor structure was found to be more appropriate for the Kenyan adolescent population. This finding is consistent with a previous study conducted among a non-Western population (Ye et al., 2015), but more research is needed to validate the measure in other sub-Saharan African contexts to confirm its generalizability.

Our study found that there was a significant positive correlation between curiosity and school climate, which is consistent with previous research that has demonstrated the influence of school climate on student’s motivation to learn (Thapa et al., 2013). Additionally, social support from friends, family, and significant others was positively correlated with curiosity. This finding supports those from other researchers of a relationship between the social network and curiosity (Thomas and Vinuales, 2017). Lastly, the fact that curiosity was significantly predicted by anxiety adds to recent research which revealed that COVID-19 restrictions may have affected the relationship between anxiety and interpersonal curiosity (Huang et al., 2021).

Although males had a higher mean CEI-II score than females, the difference was not statistically significant. However, an exploratory analysis of the relationship between curiosity and the study variables showed that the log-odds of curiosity were predicted to be 2.74 times greater in males than in females. Additionally, an increase in social support from family, significant others, and school climate was significantly associated with higher levels of curiosity among students.

In conclusion, our study supports the use of the CEI-II as a valid and reliable measure of curiosity among Kenyan adolescents. Generally, curiosity was measured to be higher among males in this population. Additionally, this study provides evidence for a positive association between curiosity and social support from family and significant others as well as school climate. This study also provides evidence for a positive relationship between curiosity and anxiety.

## 5 Limitations and future directions

Our study has several limitations. Firstly, the sample of adolescents that participated in the study was limited to those residing in one low-income area of Kenya, and therefore may not be socio- representative of the wider Kenyan adolescent population. As a result, the generalizability of our findings to other populations, such as sub-Saharan African adolescents, Kenyan adolescents, or adolescents from other non-Western contexts, may be limited. Furthermore, it is possible that the structure and function of curiosity among other non-Western adolescents may differ from those observed in this study.

Future research on other sub-Saharan African populations could potentially uncover intriguing differences in factor structure and correlations between curiosity and other constructs. As the participants in this study were secondary school students, it is possible that the level of curiosity and its associations with different outcomes could differ significantly in another setting. Future studies with sub-Saharan African populations should also test the reliability and validity of this measure in non-student populations. Lastly, it is important to note that the correlations between curiosity and other constructs were examined using a cross-sectional design, thus, limiting causal inferences.

Overall, the present study provides initial evidence for the reliability and validity of the CEI-II among a sample of Kenyan adolescents. However, future research should aim to expand on these findings by validating the measure with other sub-Saharan African populations, using a longitudinal design to determine causal relationships, examining the relationship between curiosity and academic performance, and developing an indigenous curiosity measure based on local concepts of curiosity. Such efforts will advance our understanding of curiosity and its role in the psychosocial development of sub-Saharan African adolescents.

## Data availability statement

The original contributions presented in the study are publicly available. This data can be found here: https://osf.io/z7jtd/.

## Ethics statement

This study was approved by Kenyatta University Ethical Review Committee. The study was conducted in accordance with the local legislation and institutional requirements. Written informed consent for participation was provided by minor participants’ legal guardians/next of kin. Written informed assent or consent was obtained from participants upon enrollment.

## Author contributions

NJ and TO: conceptualization. DN and TO: analysis. NJ, DN, and NK: original draft manuscript. TO, DN, NJ, and NK: edits of draft manuscript. All authors contributed to the article and approved the submitted version.
